# Recurrent Exertion‐Triggered Transient Bilateral Lower‐Limb Weakness During a Marathon in a Trained Endurance Athlete

**DOI:** 10.1002/ccr3.72951

**Published:** 2026-07-13

**Authors:** Florian Godard

**Affiliations:** ^1^ Emergency Department Centre Hospitalier Intercommunal Marmande‐Tonneins Marmande France; ^2^ Faculty of Medicine University of Lorraine Nancy France

**Keywords:** endurance athlete, exercise‐induced weakness, lower‐limb weakness, marathon, sports medicine, transient neurological symptoms

## Abstract

Recurrent, fully reversible bilateral lower‐limb weakness may occur during extreme endurance exercise in the absence of structural spinal or vascular pathology. Recognition of this rare presentation is important to avoid misdiagnosis while ensuring appropriate exclusion of serious neurological or vascular causes.

## Graphical abstract

Running pace profile during the marathon illustrating abrupt reductions in running pace associated with recurrent transient bilateral lower‐limb weakness.

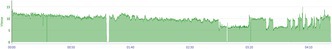

## 1 Introduction

Transient neurological symptoms occurring exclusively during prolonged endurance exercise are uncommon and diagnostically challenging. Structural spinal cord infarction and iliac artery endofibrosis represent recognized causes of exercise‐limited neurological or vascular symptoms in athletes; however, these conditions typically produce persistent deficits or unilateral findings.

Fully reversible, effort‐dependent bilateral motor deficits without identifiable structural abnormalities remain poorly characterized. We report a reproducible episode of transient bilateral lower‐limb weakness occurring during a marathon in a trained endurance athlete, with complete recovery and normal neurological and vascular investigations.

## 2 Case History/Examination

A 35‐year‐old male endurance athlete with no cardiovascular risk factors and no prior neurological history participated in the Athens Marathon in November 2021. One month earlier he had completed the Paris Marathon in 4 h 4 min without neurological symptoms. He had recently achieved personal best times in multiple road races including 10 km, 20 km, and half‐marathon distances.

Several months before the event he had also completed a 5000 km cycling journey from France to the North Cape in Norway over 40 days without limitation or neurological complaint.

Early during the marathon he developed acute diarrhea requiring a brief stop at approximately kilometer 14–15. Symptoms resolved spontaneously and did not recur during the race.

Weather conditions during the marathon were moderate (13°C, humidity 89%), and hydration was performed regularly during the race.

At approximately kilometer 36, during a downhill segment, he developed abrupt, painless, symmetric bilateral lower‐limb motor deficit that made running impossible but preserved walking ability.

There was no paraesthesia, numbness, back pain, saddle anesthesia, sphincter disturbance, or alteration of consciousness.

After several minutes of walking, he was again able to resume running normally.

However, the same deficit recurred reproducibly after a few minutes of running and again resolved completely with walking. This cycle repeated several times until the finish, preventing performance at his expected pace.

Post‐race temperature was normal. Neurological examination performed after cessation of exercise was entirely normal.

**FIGURE 1:**
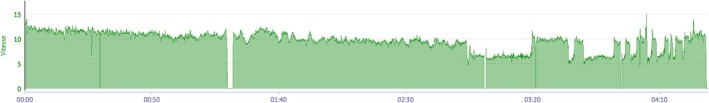
Running pace profile during the marathon derived from activity data. An early interruption corresponds to exertional diarrhea. Recurrent abrupt pace reductions after kilometer 36 correspond to transient bilateral lower‐limb motor weakness with immediate recovery during walking.

The running pace profile corresponding to these episodes is illustrated in Figure 1.

## 3 Differential Diagnosis, Investigations and Treatment

Potential differential diagnoses included spinal cord infarction, iliac artery endofibrosis, chronic exertional compartment syndrome, and other structural spinal or peripheral neurological disorders.

Magnetic resonance imaging of the spine showed no compressive, ischemic, or inflammatory lesion. A pre‐existing syringomyelia was noted but showed no imaging features suggesting acute change or clinical correlation.

Computed tomography angiography demonstrated no iliac or aorto‐iliac arterial abnormality. Duplex ultrasound of the lower limbs was normal at rest. Specialist vascular assessment at a referral center excluded iliac artery endofibrosis.

Routine laboratory investigations were unremarkable except for Epstein–Barr virus serology compatible with recent infection.

Serum potassium level obtained 8 days before the marathon was within normal limits (4.0 mmol/L on November 6, 2021). Thyroid function tests were also normal.

No specific treatment was initiated.

## 4 Outcome and Follow‐Up

No recurrence of neurological symptoms occurred during follow‐up, including during subsequent endurance training and competition. The patient progressively resumed normal athletic activity without limitation.

## 5 Discussion

This case describes a reproducible, exertion‐dependent, fully reversible bilateral lower‐limb motor deficit occurring during prolonged endurance exercise in the absence of identifiable structural pathology. The strict relationship with intense running, bilateral symmetry, and complete recovery at rest argues strongly against fixed neurological lesions.

Large cohort studies demonstrate that spinal cord infarction typically presents with persistent neurological deficits and corresponding imaging abnormalities [1]. Rare cases of exertion‐induced spinal cord infarction have been described; however, these reports consistently involve sustained deficits with radiological confirmation of ischemic injury [2]. In contrast, the complete reversibility and normal imaging in the present case argue strongly against established spinal cord infarction.

Iliac artery endofibrosis represents a recognized cause of exercise‐limited lower‐limb symptoms in endurance athletes [3]. This condition typically produces unilateral claudication‐type symptoms associated with measurable flow limitation. The bilateral painless motor deficit observed in this case, together with normal vascular imaging and specialist evaluation, makes this diagnosis unlikely.

Although hypokalemic periodic paralysis was considered as a differential diagnosis, several clinical features were atypical, including the extremely short duration of symptoms, preserved ambulation, and recovery after brief walking periods.

Exercise‐induced splanchnic hypoperfusion is well documented during prolonged endurance activity and reflects redistribution of blood flow away from the gastrointestinal circulation [4]. The early episode of exertional diarrhea observed in this case may represent such a phenomenon. Although speculative, a similar transient perfusion imbalance affecting spinal motor pathways during extreme exertion could theoretically contribute to reversible neurological symptoms.

To our knowledge, fully reversible, reproducible bilateral motor weakness occurring exclusively during endurance exercise without structural abnormalities has not been clearly characterized in the literature.

## Author Contributions

**Florian Godard:** conceptualization, data curation, investigation, writing – original draft, writing – review and editing.

## Funding

The author has nothing to report.

## Ethics Statement

The author has nothing to report.

## Consent

The patient described in this report is the author of the manuscript. Written informed consent for publication of the clinical details and accompanying figure was obtained.

## Conflicts of Interest

The author declares no conflicts of interest.

## Data Availability

Data sharing is not applicable to this article as no new datasets were generated or analysed.
